# Effectiveness of Organ Donation Information Campaigns in Germany: A Facebook Based Online Survey

**DOI:** 10.2196/ijmr.4287

**Published:** 2015-07-28

**Authors:** Tobias Terbonssen, Utz Settmacher, Christine Wurst, Olaf Dirsch, Uta Dahmen

**Affiliations:** ^1^ Experimental Transplantation Surgery Department of General, Visceral and Vascular Surgery University Hospital Jena Jena Germany; ^2^ Department of General, Visceral and Vascular Surgery University Hospital Jena Jena Germany; ^3^ Institute for Pathology Klinikum Chemnitz GmbH Chemnitz Germany

**Keywords:** organ donation, information campaign, knowledge, Germany, education

## Abstract

**Background:**

The German transplantation system is in a crisis due to a lack of donor organs. Information campaigns are one of the main approaches to increase organ donation rates. Since 2012, German health insurance funds are obliged by law to inform their members about organ donation. We raised the hypothesis: The willingness to sign a donor card rises due to the subsequent increase of specific knowledge by receiving the information material of the health insurance funds.

**Objective:**

The objective of the study was to assess the influence of information campaigns on the specific knowledge and the willingness to donate organs.

**Methods:**

We conducted an online survey based on recruitment via Facebook groups, advertisements using the snowball effect, and on mailing lists of medical faculties in Germany. Besides the demographic data, the willingness to hold an organ donor card was investigated. Specific knowledge regarding transplantation was explored using five factual questions resulting in a specific knowledge score.

**Results:**

We recruited a total of 2484 participants, of which 32.7% (300/917) had received information material. Mean age was 29.9 (SD 11.0, median 26.0). There were 65.81% (1594/2422) of the participants that were female. The mean knowledge score was 3.28 of a possible 5.00 (SD 1.1, median 3.0). Holding a donor card was associated with specific knowledge (*P*<.001), but not with the general education level (*P*=.155). Receiving information material was related to holding a donor card (*P*<.001), but not to a relevant increase in specific knowledge (difference in mean knowledge score 3.20 to 3.48, *P*=.006). The specific knowledge score and the percentage of organ donor card holders showed a linear association (*P*<.001).

**Conclusions:**

The information campaign was not associated with a relevant increase in specific knowledge, but with an increased rate in organ donor card holders. This effect is most likely related to the feeling of being informed, together with an easy access to the organ donor card.

## Introduction

### Lack of Donors in German Transplantation System

The German transplantation system is in a crisis due to a lack of donor organs. About 12,000 patients are waiting for an organ graft [1]. Every year more than 1000 patients in Germany die because they cannot be supplied with an organ graft in time [2].

Organ donation rates in Germany decreased constantly over the last few years. In 2012, there were only 1046 deceased organ donors. These were 12.8% fewer donors compared to 2011, and it is the lowest number of organ donors since 2003 [3]. In 2013, this number decreased again to only 876 deceased organ donors [4]. The number of new registrations on the organ transplant waiting list increased from 8264 patients in 2004 to 10,106 patients in 2013 [5]. These two opposing developments are aggravating the lack of donor organs, creating a dramatic situation.

### Information Campaigns Used to Increase Organ Donation Rates

Information campaigns are one of the main approaches to increase organ donation rates [6]. Organ donation information campaigns are dedicated to attract the attention of the targeted audience to the issue. Once the attention is directed toward organ donation, the distributed information material should help the recipients to make a balanced decision based on the presumingly increased personal level of specific knowledge. In November 2012, the German Transplant Act was updated to support this approach. Since then, health insurance funds are obliged to inform their members over 16 years of age about organ donation. The information has to be provided in an objective manner. The information material has to be distributed every two years including an organ donor card form. However, there is no obligation for the members to fill and sign an organ donor card [7].

Generally, educational information campaigns have the potential to improve the willingness to donate [8-16]. Still, the effect of this nationwide information campaign toward specific knowledge concerning organ donation needs to be quantified using an objective knowledge score. We wanted to know the answers to the following questions. What is the effect of conducting information campaigns by unsolicitedly distributing written information (flyers) to the population? Does distribution of written information lead to actual reading and processing of the information, and ultimately to an increased declarative knowledge of the participants? Does this distribution lead to an increase in organ donor card holders? Is an increase in knowledge leading to an increase in organ donor card holders? We raised the hypothesis: The willingness to sign a donor card rises due to the subsequent increase of specific knowledge by receiving the information material of the health insurance funds.

## Methods

### Survey

The aim of this study was to evaluate the influence of a nationwide information campaign on the willingness to sign a donor card. The information campaign was conducted by the health insurance agencies, as enforced by law. We conducted an open Internet survey from June 10 to July 18, 2013 using soscisurvey.de as the questionnaire tool. Our target population was the general population between 15 and 64 years of age. An institutional review board (IRB) approval was not necessary (decision of the IRB of the University Hospital Jena). The questionnaires were anonymous, and we did not save any personal data. On the first screen, participants were told that the questionnaire would take 10 minutes. We did tell the topic of the survey, but we did not tell its purpose to avoid bias. The investigators and their contact details have been displayed. The questionnaire design was based on the literature of Kuckartz, Porst, and Raab-Steiner [17-19]. The survey comprised 44 items. A maximum of 10 items per screen were distributed over 16 screens. The participants were able to change their answers through a “Back” button. There was no review step displaying a summary. We did not use cookies, and did not save the participants Internet protocol address. In conclusion, theoretically, participants were able to participate more than once. Questionnaires that terminated early were also analyzed. We did not exclude questionnaires due to atypical timestamps. We performed a pretest and distributed the questionnaire in the revised final version. The survey questionnaire is appended as multimedia appendix (see Multimedia Appendix 1).

The hyperlink to the survey was distributed via 202 Facebook groups of all kinds. To avoid bias, we did not use any organ donation group or thematically similar groups. In order to take advantage of the so-called “snowball effect” [20], we recommended the users to share the hyperlink via Facebook. A table of all Facebook groups is appended as multimedia appendix (see Multimedia Appendix 2). In addition, we used Facebook advertisement that was shown 141,366 times to different Facebook users. The hyperlink was also distributed using mailing lists of medical faculties in Germany.

To explore the specific knowledge concerning organ donation, five factual questions with different levels of difficulty were asked, see Table 1. The following response options were offered: two false answers, the correct answer, and "I don't know". To avoid bias, these four response options were mixed randomly for every single questionnaire. A sum of 0-5 correctly answered questions could be achieved resulting in the “specific knowledge score”. This new variable was taken as a marker for the individual knowledge concerning organ donation.

At the time of the survey, some, but not all, health insurance agencies had already sent their information material to their members [21]. Therefore, it was possible to compare two different groups. We call participants prior to receiving information-material “uninformed participants”. Participants after receiving the material are “informed participants”. The group of organ donor card holders among “uninformed participants” was taken as the control group to explore the effect of the educational intervention. The relative difference in organ donor card holders was considered to be the effect of the information campaign.

**Table 1:** Exploration of specific knowledge using five factual questions, organized by level of difficulty.

Question	Response options^a^	n (%)
Which organ can NOT be donated?	Brain	641/650 (98.6)
	Kidney	1/650 (0.2)
	Liver	4/650 (0.6)
	I don’t know.	4/650 (0.6)
Which statement is correct?	Physicians and relatives have to stick to the entries in organ donor cards.	529/649 (81.5)
	The organ donor card is registered at organ donation agency and the entries are recorded.	77/649 (11.8)
	Organ donor card holders get themselves an organ faster when they are sick.	14/649 (2.1)
	I don’t know.	30/649 (4.6)
Which statement is correct?	From the age of 16 years, minors can state their willingness in an organ donor card.	501/648 (77.3)
	When your attitude toward organ donation changes, you have to inform the public health office.	33/648 (5.1)
	Before the completion of an organ donor card, a thorough examination from a physician is necessary.	61/648 (9.4)
	I don’t know.	53/648 (8.2)
How long the organ recipient usually has to take drugs after the organ transplantation?	A lifetime.	328/651 (50.4)
	Until the organ was accepted by the recipient’s body.	165/651 (25.3)
	Until the organ reached its entire function.	91/651 (13.9)
	I don’t know.	68/651 (10.4)
Which of these drugs is usually NOT used during organ transplantations?	Acetylcysteine	136/649 (20.9)
	Cyclosporine	20/649 (3.0)
	Mycophenolate	34/649 (5.2)
	I don’t know.	460/649 (70.9)

^a^The correct answers are underlined. The relative proportion of participants’ responses is given for every question.

### Statistics

Due to our distribution method, we assumed a distinct overrepresentation of participants of the medical sector. To minimize this bias, we weighted the percentage of the medical sector to realistic 9.52%. We calculated this percentage based on the following numbers: In 2012, 54,154,000 inhabitants age between 15 and 64 years (our target population) lived in Germany, of whom about 5,155,000 inhabitants worked in the medical sector [22,23].

Descriptive statistical analysis was carried out. We compared the different quantitative variables using Student’s t test or Mann–Whitney U test, qualitative variables using chi-square test. P values < .05 were considered statistically significant.

Statistical analyses were performed with IBM SPSS Statistics 21.0 for Windows (IBM Corporation).

## Results

### Overrepresentation of Academics, Participants of the Medical Sector, and Younger Participants

A total of 2484 participants took part in our survey. There were 65.81% (1594/2422) that were female. The youngest respondent was 14 and the oldest 77 years old (mean age 29.9, SD 11.0, median 26.0, interquartile range 22-35). Participants from all educational levels were reached with our survey, albeit with an overrepresentation of high education compared with a statistic from the German Federal Statistical Office [24]. However, the statistical comparison of the epidemiological data did not reveal any significant difference between the “informed” and “uninformed” population (Table 2). As expected, we found an overrepresentation of participants from the medical sector (62.49%, 1533/2453). If not stated otherwise, all values are presented based on a percentage of participants of the medical sector weighted to 9.52%.

**Table 2:** Comparison of epidemiological variables between “informed” and “uninformed” participants.

Epidemiological variable		“informed” participants	“uninformed” participants
**Age**			
	Mean (SD)	31.07 (11.38)	29.70 (10.86)
	Median	27	26
**Sex, n (%)**			
	Female	177/294 (60.2)	370/589 (62.8)
	Male	117/294 (39.8)	219/589 (37.2)
**Level of education, n (%)**			
	None (including secondary school graduation)	1/299 (0.3)	3/616 (0.5)
	Hauptschulabschluss (secondary general school certificate)	14/299 (4.7)	37/616 (6.0)
	Mittlere Reife (secondary school graduation)	38/299 (12.7)	71/616 (11.5)
	Completed apprenticeship	45/299 (15.1)	87/616 (14.1)
	Fachabitur (entrance qualification for studying at a university of applied sciences)	28/299 (9.4)	46/616 (7.5)
	Abitur (university-entrance diploma)	118/299 (39.5)	254/616 (41.2)
	University degree	48/299 (16.1)	95/616 (15.4)
Working in medical sector, n (%)		31/300 (10.3)	57/617 (9.2)

### Association of Holding a Donor Card With Specific Knowledge

The rate of donor card holders was correlated with specific knowledge. The overall population reached a mean knowledge score of 3.28 (SD 1.10, median score 3.0, range 2-5, interquartile range 3-4). The specific knowledge score and the percentage of organ donor card holders showed a linear association (*P*<.001): 12% (5/41) of participants who reached 1 point in the specific knowledge score carried an organ donor card. There were 27% (25/92) of participants who reached 2 points that carried an organ donor card. There were 54.4% (124/228) of participants who reached 3 points that carried an organ donor card. There were 70.7% (130/184) of participants who reached 4 points that carried an organ donor card, and 74% (64/87) of participants who reached 5 points that did so.

We compared the participants of the medical sector with the ones of the general population. There were 76.70% (1149/1498) of the participants of the medical sector that did hold an organ donor card, whereas the percentage in the general population was lower (51.2%, 454/886, *P*<.001). These values are based on the unweighted percentage of participants of the medical sector. In addition, we did not find a working sector with more organ donor card holders than in the medical sector.

Holding an organ donor card was not correlated to the level of education. There were 54.9% (426/776) of participants who had a level of education similar or higher than a completed apprenticeship or a Fachabitur (entrance qualification for studying at a university of applied sciences) that signed an organ donor card. There were 49.0% (100/204) of the group of participants who had a lower level of education that signed an organ donor card. These differences were not statistically significant (*P*=.155).

Due to the correlation between specific knowledge and holding an organ donor card, knowledge campaigns should be intensified!

### Association of Receiving Information Material With Holding a Donor Card

Association between receiving information material of the health insurance funds and specific knowledge is very slight. In the “informed” group, the mean specific knowledge score was 3.48 (SD 1.01, median 3.0, interquartile range 3-4). Compared to the “uninformed” group, we found no relevant difference (mean knowledge score 3.20, SD 1.1, median 3.0, interquartile range 3-4) (*P*=.006).

However, receiving information material of the health insurance funds was correlated with holding an organ donor card. There were 32.7% (300/917) of the participants that stated to have received information material from their health insurance fund. A high proportion of 68.1% (194/285) of them carried a donor card, whereas only 46.9% (281/599) of the “uninformed” group did hold a donor card (*P*<.001). The odds ratio for holding a donor card was 2.41 (1.79-3.24). Providing information together with an organ donor card was associated with a 20% difference in donor card holders.

Reading the information material of the health insurance funds was also correlated with holding an organ donor card. We divided the number of the participants who received information material into the ones who had read the material and the ones who had not. The majority of participants, 78.8% (237/301), stated to have actually read the material. More than two thirds (71.7%, 160/223) of this interested and active subgroup had signed a donor card, which is significantly higher (*P*<.001) than in the group who did not read it (55%, 34/62). We found an odds ratio for holding a donor card of 2.09 (1.17-3.73).

These results indicate that receiving information material leads to a higher percentage of organ donor card holders. Actually reading this material leads to an essential increase in the percentage.

## Discussion

### Principal Findings

Our survey used the unique opportunity of conducting a study on a nationwide intervention without intervening by us.

### Information Campaigns Lead to More Organ Donor Card Holders

The two groups of participants did not show any differences in age, gender, working sector, or level of education. Therefore, we attributed the observed difference in organ donor card holders in the “informed group” to the “uninformed group” to the educational intervention of the health insurance funds.

A study of Techniker Krankenkasse revealed that 31% of health insurance policyholders of this particular health insurance fund were donor card holders, compared to 21% among the general population. This health insurance fund was the only one that had sent information material to its members at that time [25]. This was a first hint that the information campaign of the health insurance funds was successful, and it matches our findings. Our results indicate that receiving information material leads to a higher percentage of organ donor card holders. Actually reading this material leads to an essential increase in the percentage. Several publications indicate the potential of information campaigns to increase the number of organ donor card holders [8-16].

On the contrary, a study by Radunz et al did not show significant differences in the number of organ donor card holders after educational interventions with medical students. With 67% before the intervention, there were already a high proportion of donor card holders among the participants [26]. See Multimedia Appendix 3 for a table containing literature of educational interventions on organ donation and their results.

### Greater Knowledge Concerning Organ Donation Leads to More Organ Donor Card Holders

We could also demonstrate that greater knowledge concerning organ donation was correlated to holding an organ donor card. Comparable to our results, several publications indicate that knowledge regarding organ donation was a significant factor for increased willingness to donate [27-33]. See Multimedia Appendix 4 for a table containing literature of the correlation between knowledge about organ donation and the willingness to donate.

We were able to demonstrate that participants with a medical background or working in the medical sector were more likely to hold an organ donor card than participants from other working sectors. A study on medical students by Gauher et al showed that the medical students were more likely to donate than other students due to their greater knowledge concerning organ donation [34]. Another study by McGlade and Pierscionek on student nurses found that improved knowledge leads to more positive discussion behavior of student nurses about organ donation [35]. Hobeika et al found contrary results. In a study with medical students and surgeons, they discovered that participants with less professional experience are more willing to agree to organ donation. Especially responders who had witnessed a procurement procedure showed more refusal to donate their organs [36].

Our findings demonstrate no significant correlation between the level of education and holding an organ donor card. Yilmaz found similar results [10], whereas Boulware et al found that participants with higher education level and more income were more willing to become an organ donor than participants with less education and income [37].

### Information Campaign Did Not Lead to Greater Knowledge

Several publications indicate that education interventions have the potential to increase the specific knowledge concerning organ donation [14,38-40].

Therefore, one could assume that the increase in the percentage of organ donor card holders was due to a greater knowledge because of the information campaigns. Our results show that this increase in knowledge was very slight, and it presumably was not decisive for the increase in the percentage of organ donor card holders. A discussion about the true reasons for this increase might be speculative. Most likely the key reason is that an organ donor card form was enclosed to the information material [7]. Offer of information and ease to fill the form were coming together and did facilitate the decision and the written documentation of this decision.

### Limitations

It is possible that five factual questions were not sufficient to clarify the effect of the information material on specific knowledge concerning organ donation. Future examinations should verify the effect by using a questionnaire only containing factual questions.

Our study indicates a basic level of 46.9% (281/599) organ donor card holders in our sample group. This is much more than in a representative previous study (21%) [25]. These different findings must not be related to an increase in the over-all willingness to donate organs, but may be explained by the self-selection bias. Even though we strictly refrained from mentioning the topic of the survey while distributing the hyperlink, people with more interest in organ donation were presumably more likely to participate. We used Facebook for distributing the hyperlink. This procedure is controversial because Facebook does not represent the whole population. Nevertheless, over 25 million Germans visit their Facebook profile every month. These are nearly half of all German Internet users [41]. Furthermore, Nelson as well as Fenner concluded that using social media sites such as Facebook was a successful way in recruiting participants for surveys [42,43]. Baltar and Brunet got the same conclusion, especially with the snowball sampling method using Facebook [20].

### Conclusions

The information campaign was not associated with an increase in specific knowledge, but still with an increased rate in organ donor card holders. This effect is most likely related to the feeling of being informed together with an easy access to the organ donation card. Future educational interventions should put an extra effort toward increasing the knowledge in order to maximize the effect. Special efforts should be undertaken to improve the knowledge on how to become an organ donor 44]. Furthermore, information campaigns comparable to the campaigns of the health insurance funds should be repeated periodically. In addition, information about organ donation should be provided in more ways, as lessons in school, brochures in public buildings, or in television shows. Moreover, the access to organ donor card forms should be improved. These cards should be displayed at public buildings and additionally sent to every household every few years.

## Appendix

Multimedia Appendix 1 The survey questionnaire.

Multimedia Appendix 2 Used Facebook groups.

Multimedia Appendix 3 Literature of educational interventions on organ donation and their results.

Multimedia Appendix 4 Literature of the correlation between knowledge about organ donation and the willingness to donate.

## Abbreviations

IRB: institutional review board

## References

[1] Tuffs A (2011). Germany pushes for more organ donation. BMJ.

[2] Wesslau K, Grosse Katharina, Krüger Ronald, Kücük Onur, Mauer Dietmar, Nitschke Frank-Peter, Norba Daniela, Manecke Axel, Polster Frank, Gabel Doris (2007). How large is the organ donor potential in Germany? Results of an analysis of data collected on deceased with primary and secondary brain damage in intensive care unit from 2002 to 2005. Transpl Int.

[3] Deutsche Stiftung Organtransplantation (2012). Annual Report 2012.

[4] Deutsche Stiftung Organtransplantation Annual Report 2013.

[5] Eurotransplant (2015). Eurotransplant.

[6] Heuer M, Remmer N, Radünz S, Frühauf N R, Canbay A, Paul A, Kaiser GM (2013). Disposition for organ donation: Analysis of a survey and trial of 974 respondents. Zentralbl Chir.

[7] Deutscher Bundestag (2012). Bundesgesetzblatt Teil I(33).

[8] Heuer M, Radunz S, von HF, Kirchner C, Wittenburg N, Stammen K, Paul A, Kaiser G (2014). Online intervention study--Willingness to donate organs among the employees of a German University. Eur J Med Res.

[9] Rey JW, Grass V, Galle PR, Werner C, Hoffman A, Kiesslich R, Hammer GP (2013). Education in organ donation among students in Germany - results of an intervention study. Ann Transplant.

[10] Yilmaz TU (2011). Importance of education in organ donation. Exp Clin Transplant.

[11] Frates J, Bohrer GG, Thomas D (2006). Promoting organ donation to Hispanics: The role of the media and medicine. J Health Commun.

[12] Smits M, Dijker AJ, Ryckman RM, van den Borne Bart (2006). Increasing Dutch adolescents' willingness to register their organ donation preference: The effectiveness of an education programme delivered by kidney transplantation patients. Eur J Public Health.

[13] Tokalak I, Kut A, Moray G, Emiroglu R, Erdal R, Karakayali H, Haberal M (2006). Knowledge and attitudes of high school students related to organ donation and transplantation: A cross-sectional survey in Turkey. Saudi J Kidney Dis Transpl.

[14] Reubsaet A, Brug J, Nijkamp MD, Candel M J J M, van Hooff J P, van den Borne H W (2005). The impact of an organ donation registration information program for high school students in the Netherlands. Soc Sci Med.

[15] Piccoli GB, Soragna G, Putaggio S, Burdese M, Longo P, Rinaldi D, Bergamo D, Mezza E, Consiglio V, Novaresio C, Giacchino F, Jeantet A, Segoloni GP (2004). Efficacy of an educational program on dialysis, renal transplantation, and organ donation on the opinions of high school students: A randomized controlled trial. Transplant Proc.

[16] D'Alessandro AM, Peltier JW, Phelps JE (2008). Increasing organ donations after cardiac death by increasing DCD support among health care professionals: A case report. Am J Transplant.

[17] Kuckartz U, Ebert Ta, Rädiker S, Stefer C (2009). Evaluation online: Internetgestützte Befragung in der Praxis (German Edition).

[18] Porst R (2011). Fragebogen: Ein Arbeitsbuch (Studienskripten zur Soziologie) (German Edition).

[19] Raab-Steiner E, Benesch M (2012). Der Fragebogen: Von der Forschungsidee zur SPSS/PASW Auswertung. 3rd ed.

[20] Baltar F, Brunet I (2012). Social research 2.0: Virtual snowball sampling method using Facebook. Internet Research.

[21] aerzteblatt (2013). Organspende: So informieren die Krankenkassen.

[22] Statistisches Bundesamt.

[23] Statistisches Bundesamt.

[24] Statistisches Bundesamt.

[25] Techniker Krankenkasse.

[26] Radunz S, Juntermanns B, Heuer M, Frühauf Nils R, Paul A, Kaiser GM (2012). The effect of education on the attitude of medical students towards organ donation. Ann Transplant.

[27] Thornton JD, Wong KA, Cardenas V, Curtis JR, Spigner C, Allen MD (2006). Ethnic and gender differences in willingness among high school students to donate organs. J Adolesc Health.

[28] Febrero B, Ríos A, Martínez-Alarcón L, López-Navas A, Almela J, Sánchez J, Ramis G, Ramírez P, Parrilla P (2013). Information received by secondary school teaching personnel about organ donation and transplantation: A study in the southeast of Spain. Transplant Proc.

[29] Febrero B, Ríos Antonio, López-Navas Ana, Martínez-Alarcón Laura, Almela J, Sánchez Álvaro, Sánchez Jose, Parrilla JJ, Ramírez Pablo, Parrilla P (2014). A multicenter study of the attitude of secondary school teachers toward solid organ donation and transplantation in the southeast of Spain. Clin Transplant.

[30] Trompeta JA, Cooper BA, Ascher NL, Kools SM, Kennedy CM, Chen J (2012). Asian American adolescents' willingness to donate organs and engage in family discussion about organ donation and transplantation. Prog Transplant.

[31] Wong LP (2011). Knowledge, attitudes, practices and behaviors regarding deceased organ donation and transplantation in Malaysia's multi-ethnic society: A baseline study. Clin Transplant.

[32] Al-Ghanim SA (2009). The willingness toward deceased organ donation among university students. Implications for health education in Saudi Arabia. Saudi Med J.

[33] Saleem T, Ishaque S, Habib N, Hussain SS, Jawed A, Khan AA, Ahmad MI, Iftikhar MO, Mughal HP, Jehan I (2009). Knowledge, attitudes and practices survey on organ donation among a selected adult population of Pakistan. BMC Med Ethics.

[34] Gauher ST, Khehar R, Rajput G, Hayat A, Bakshi B, Chawla H, Cox BM, Warrens AN (2013). The factors that influence attitudes toward organ donation for transplantation among UK university students of Indian and Pakistani descent. Clin Transplant.

[35] McGlade D, Pierscionek B (2013). Can education alter attitudes, behaviour and knowledge about organ donation? A pretest-post-test study. BMJ Open.

[36] Hobeika MJ, Simon R, Malik R, Pachter HL, Frangos S, Bholat O, Teperman S, Teperman L (2009). U.S. surgeon and medical student attitudes toward organ donation. J Trauma.

[37] Boulware LE, Ratner LE, Sosa JA, Cooper LA, LaVeist TA, Powe NR (2002). Determinants of willingness to donate living related and cadaveric organs: Identifying opportunities for intervention. Transplantation.

[38] Rachmani R (2000). The organ donation process-workshop. Transplant Proc.

[39] Wig N, Aggarwal P, Kailash S, Handa R, Wali JP (1999). Awareness of brain death and organ transplantation among high school children. Indian J Pediatr.

[40] Reville P, Zhao C, Perez T, Nowacki AS, Phillips D, Bowen G, Starling N, Pflaum B, Strickland R, Fung J, Askar M (2013). A student leadership model for promoting educational programs in organ donation and transplantation. Transplant Proc.

[41] Kulow T Facebook.

[42] Nelson EJ, Hughes J, Oakes JM, Pankow JS, Kulasingam SL (2014). Estimation of geographic variation in human papillomavirus vaccine uptake in men and women: An online survey using Facebook recruitment. J Med Internet Res.

[43] Fenner Y, Garland SM, Moore EE, Jayasinghe Y, Fletcher A, Tabrizi SN, Gunasekaran B, Wark JD (2012). Web-based recruiting for health research using a social networking site: An exploratory study. J Med Internet Res.

[44] D'Alessandro AM, Peltier JW, Dahl AJ (2012). The impact of social, cognitive and attitudinal dimensions on college students' support for organ donation. Am J Transplant.

