# Supplementary material for: Effectiveness of Organ Donation Information Campaigns in Germany: A Facebook Based Online Survey
Source: Interact J Med Res. 2015 Jul 28;4(3):e16. doi: 10.2196/ijmr.4287 (PMC4705356; doi:10.2196/ijmr.4287)

Universitätsklinikum Jena

0% ausgefüllt

Organspende - Find' ich gut?

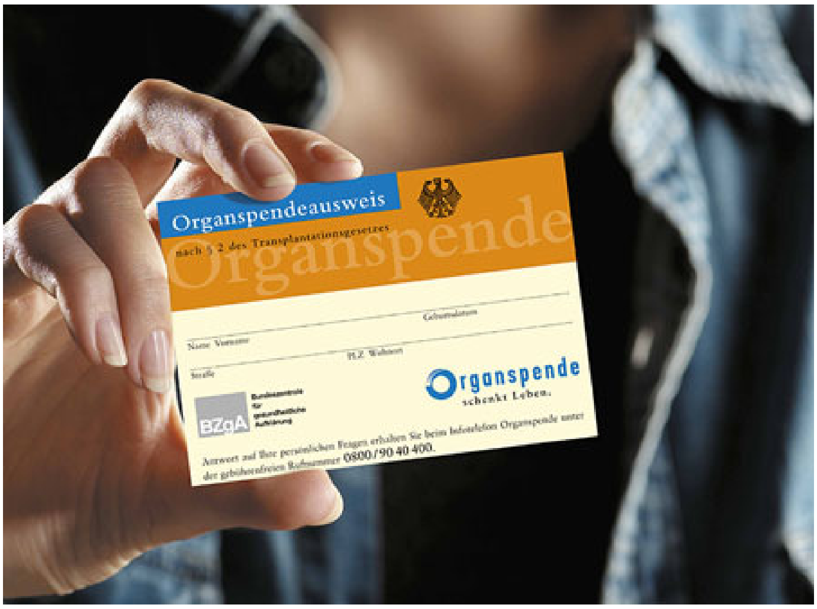

© Bundeszentrale für gesundheitliche Aufklärung

Wir freuen uns, dass Sie sich dazu bereit erklären, an unserer Umfrage zur Organspende teilzunehmen!

Mit dieser Umfrage wollen wir die Einstellung von jungen Erwachsenen zum Thema Organspende erfragen.
Die Beantwortung des Fragebogens wird ca. 10 Minuten dauern. Wenn Sie Fragen zu unserer Umfrage haben, so können Sie sich gerne jederzeit an uns wenden.

Um mit der Umfrage zu beginnen, klicken Sie bitte unten auf "Weiter"!

Experimentelle Transplantationschirurgie
Klinik für Allgemein-, Viszeral- und Gefäßchirurgie
Universitätsklinikum Jena
Erlanger Allee 101
07747 Jena

Internetauftritt

Ansprechpartner:
Tobias Terbonßen
E-Mail: tobias.terbonssen@uni-jena.de

Betreuerin:
Prof. Dr. Uta Dahmen
E-Mail: uta.dahmen@med.uni-jena.de

mit freundlicher Unterstützung von

AOK PLUS

Biotest – From Nature for Life

Weiter

8% ausgefüllt

1. Besitzen Sie einen Organspendeausweis?

Bitte wählen Sie die zutreffende Option aus!

- **Ich besitze einen ausgefüllten Organspendeausweis und trage diesen bei mir.**
  - Ich gestatte die Organentnahme ohne Ausnahme
  - Ich gestatte die Organentnahme mit Ausnahmen
  - Ich widerspreche einer Organentnahme
  - Eine von mir angegebene Person soll darüber entscheiden
- **Ich besitze einen ausgefüllten Organspendeausweis und trage diesen NICHT bei mir.**
- **Ich besitze KEINEN ausgefüllten Organspendeausweis.**

2. Würden Sie ein Organ von einem fremden Spender annehmen, wenn Sie es zum Überleben benötigen würden?

Nein	Eher Nein	Eher Ja	Ja
○	○	○	○

3. Sind Sie zu einer Lebendspende bereit? (z.B. Leber, Niere)

Die Lebendspende bedeutet, dass Ihnen zu Lebzeiten ein Organ entnommen wird, um es einem kranken nahen Verwandten oder engen Freund zu geben.

- Ja, wenn ich einem Menschen damit helfen kann.
- Ja, aber nur bei sehr engen Angehörigen.
- Nein, grundsätzlich nicht.
- Ich habe darüber noch nicht nachgedacht.

Zurück Weiter

17% ausgefüllt

4. Bitte bewerten Sie folgende Aussagen anhand Ihrer persönlichen Einstellung.

Die nachfolgenden Fragen beziehen sich auf die Organspende **nach dem Tod!**

	Trifft überhaupt nicht zu 1	2	3	4	Trifft voll zu 5	weiß nicht
Ich habe Vertrauen in die Entscheidungen der Ärzte im Bereich der Organspende.	○	○	○	○	○	○
Mein Vertrauen in Ärzte ist durch die jüngsten Transplantationsskandale gesunken.	○	○	○	○	○	○
Ich denke, dass bei der Organvergabe reiche Menschen bevorzugt werden.	○	○	○	○	○	○
Ich habe Angst davor, bei der Organentnahme Schmerzen zu verspüren.	○	○	○	○	○	○
Ich habe Angst davor, dass ich bei der Organentnahme noch gar nicht wirklich tot bin.	○	○	○	○	○	○
Mir gefällt die Idee, dass nach meinem Tod wenigstens einzelne Organe eine Zeit lang weiterleben könnten.	○	○	○	○	○	○
Ich glaube, dass Organspender in manchen Krankenhäusern vorzeitig für tot erklärt werden, wenn dort dringend Organe benötigt werden.	○	○	○	○	○	○
Ich sorge mich darum, dass mein Körper nach der Organentnahme entstellt aussieht.	○	○	○	○	○	○
Meine Bereitschaft zur Organspende ist durch die Berichterstattung über dieses Thema in der letzten Zeit eher gesunken.	○	○	○	○	○	○

5. Haben Sie bereits von Ihrer Krankenkasse Informationsmaterial über Organspende zugeschickt bekommen?

- ○ **Ja, und ich habe es auch gelesen**
- ○ **Ja, ich habe es aber nicht gelesen**
- ○ **Nein**
- ○ **Weiß nicht**

Zurück   Weiter

42% ausgefüllt

Warnung (Seite 6)

Die Variable **OR07** wurde im Interview noch nicht abgefragt, daher kann die Funktion value() keinen Antwort-Wert ermitteln.

6. Haben Sie jemals einen Kurs in Erster Hilfe besucht?

Hiermit ist nicht der Kurs in Lebensrettenden Sofortmaßnahmen im Rahmen des Führerscheins gemeint!

- ○ Ja
- ○ Nein

7. Engagieren Sie sich ehrenamtlich in einem der folgenden Bereiche?

Bitte wählen Sie alle Bereiche aus, in denen Sie sich ehrenamtlich engagieren. Sie können auch **mehrere Bereiche** angeben. Wenn Sie sich nicht ehrenamtlich engagieren, so kreuzen Sie einfach keine Antwortmöglichkeit an!

- ☐ In der Kirche
- ☐ Im Sportverein
- ☐ Im Krankenhaus
- ☐ In der Pflege
- ☐ In der Politik
- ☐ In der Bildung
- ☐ Sonstiges (bitte nachfolgend angeben) ________

Zurück Weiter

50% ausgefüllt

8. Sind Sie bei der Deutschen Knochenmarkspenderdatei als Knochenmarkspender registriert?

- Ja
- Nein

9. Haben Sie schon mindestens ein Mal Blut gespendet?

- Ja
- Nein

Zurück | Weiter

58% ausgefüllt

10. Die folgenden 5 Fragen testen Ihr Wissen über die Organspende. Bitte beantworten Sie diese Fragen nur anhand Ihres Wissens und schlagen Sie nicht nach! Nur so können die Daten sinnvoll verwendet werden. Es ist völlig in Ordnung, wenn Sie nicht alle Fragen beantworten können! Bitte klicken Sie direkt auf die Antwort, die Sie für zutreffend halten. So gelangen Sie sofort zur nächsten Frage. Es kann nicht vor- oder zurückgeblättert werden!

Drücken Sie auf „Weiter zur ersten Frage" und NICHT den „Weiter"-Button!

Weiter zur ersten Frage

58% ausgefüllt

10. Die folgenden 5 Fragen testen Ihr Wissen über die Organspende. Bitte beantworten Sie diese Fragen nur anhand Ihres Wissens und schlagen Sie nicht nach! Nur so können die Daten sinnvoll verwendet werden. Es ist völlig in Ordnung, wenn Sie nicht alle Fragen beantworten können! Bitte klicken Sie direkt auf die Antwort, die Sie für zutreffend halten. So gelangen Sie sofort zur nächsten Frage. Es kann nicht vor- oder zurückgeblättert werden!

Drücken Sie auf „Weiter zur ersten Frage" und NICHT den „Weiter"-Button!

1.) Wie lange muss der Organempfänger in aller Regel Medikamente einnehmen, nachdem er das Organ bekommen hat?

- Bis das Organ vom Körper angenommen wurde
- Ich weiß es nicht
- Bis das Organ seine vollständige Funktion erreicht hat
- Lebenslang

58% ausgefüllt

10. Die folgenden 5 Fragen testen Ihr Wissen über die Organspende. Bitte beantworten Sie diese Fragen nur anhand Ihres Wissens und schlagen Sie nicht nach! Nur so können die Daten sinnvoll verwendet werden. Es ist völlig in Ordnung, wenn Sie nicht alle Fragen beantworten können! Bitte klicken Sie direkt auf die Antwort, die Sie für zutreffend halten. So gelangen Sie sofort zur nächsten Frage. Es kann nicht vor- oder zurückgeblättert werden!

Drücken Sie auf „Weiter zur ersten Frage“ und NICHT den „Weiter“-Button!

2.) Welches Organ kann NICHT gespendet werden?

- Ich weiß es nicht
- Niere
- Leber
- Gehirn

58% ausgefüllt

10. Die folgenden 5 Fragen testen Ihr Wissen über die Organspende. Bitte beantworten Sie diese Fragen nur anhand Ihres Wissens und schlagen Sie nicht nach! Nur so können die Daten sinnvoll verwendet werden. Es ist völlig in Ordnung, wenn Sie nicht alle Fragen beantworten können! Bitte klicken Sie direkt auf die Antwort, die Sie für zutreffend halten. So gelangen Sie sofort zur nächsten Frage. Es kann nicht vor- oder zurückgeblättert werden!

Drücken Sie auf „Weiter zur ersten Frage" und NICHT den „Weiter"-Button!

3.) Welche Aussage stimmt?

- Weiß nicht
- Menschen mit Organspendeausweis bekommen selber schneller ein Organ, wenn sie krank sind.
- An die Eintragungen auf dem Organspendeausweis müssen sich Ärzte und Angehörige halten.
- Der Organspendeausweis wird bei der Organspendebehörde registriert und die Eintragungen werden gespeichert.

58% ausgefüllt

10. Die folgenden 5 Fragen testen Ihr Wissen über die Organspende. Bitte beantworten Sie diese Fragen nur anhand Ihres Wissens und schlagen Sie nicht nach! Nur so können die Daten sinnvoll verwendet werden. Es ist völlig in Ordnung, wenn Sie nicht alle Fragen beantworten können! Bitte klicken Sie direkt auf die Antwort, die Sie für zutreffend halten. So gelangen Sie sofort zur nächsten Frage. Es kann nicht vor- oder zurückgeblättert werden!

Drücken Sie auf „Weiter zur ersten Frage" und NICHT den „Weiter"-Button!

4.) Welches der nachfolgenden Medikamente kommt in aller Regel NICHT im Rahmen der Transplantation zum Einsatz?

- Acetylcystein
- Mykophenolat
- Ich weiß es nicht
- Ciclosporin

58% ausgefüllt

10. Die folgenden 5 Fragen testen Ihr Wissen über die Organspende. Bitte beantworten Sie diese Fragen nur anhand Ihres Wissens und schlagen Sie nicht nach! Nur so können die Daten sinnvoll verwendet werden. Es ist völlig in Ordnung, wenn Sie nicht alle Fragen beantworten können! Bitte klicken Sie direkt auf die Antwort, die Sie für zutreffend halten. So gelangen Sie sofort zur nächsten Frage. Es kann nicht vor- oder zurückgeblättert werden!

Drücken Sie auf „Weiter zur ersten Frage" und NICHT den „Weiter"-Button!

5.) Welche der folgenden Aussagen stimmt?

- Ab einem Alter von 16 Jahren können Minderjährige ihre Bereitschaft zur Organspende im Organspendeausweis erklären.
- Vor dem Ausfüllen eines Organspendeausweises ist eine gründliche Untersuchung beim Arzt notwendig.
- Wenn sich die Einstellung zur Organspende geändert hat, muss man dies beim Gesundheitsamt melden.
- Ich weiß es nicht.

67% ausgefüllt

11. Bitte beantworten Sie die folgenden Fragen anhand Ihrer persönlichen Einstellung. Wenn Sie keine Antwort wissen, klicken Sie auf „weiß nicht".

	trifft überhaupt nicht zu 1	2	3	4	trifft voll zu 5	weiß nicht
Ich bin religiös.	○	○	○	○	○	○
Ich glaube an ein Leben nach dem Tod.	○	○	○	○	○	○
Ich bin mit meinem Leben zufrieden.	○	○	○	○	○	○
Ich bin hilfsbereit.	○	○	○	○	○	○
Ich mache viel Sport.	○	○	○	○	○	○
Ich ernähre mich gesund.	○	○	○	○	○	○

12. Bei welcher <u>gesetzlichen</u> Krankenkasse sind Sie mit Ihrer Vollversicherung krankenversichert? Oder sind Sie <u>privat</u> krankenversichert?

- ○ privat krankenversichert (PKV)
- ○ AOK – Allgemeine Ortskrankenkasse
- ○ Barmer GEK (auch: Barmer, BEK)
- ○ BKK – bei einer Betriebskrankenkasse (egal welche)
- ○ DAK – Deutsche Angestellten-Krankenkasse
- ○ IKK – bei einer Innungskrankenkasse (egal welche)
- ○ KKH (auch Kaufmännische Krankenkasse, KKH-Allianz)
- ○ Bundesknappschaft
- ○ TK – Techniker Krankenkasse
- ○ bei einer anderen gesetzlichen Krankenkasse
- ○ weiß nicht

Zurück | Weiter

75% ausgefüllt

13. In welchem Bundesland leben Sie?

- Baden-Württemberg
- Bayern
- Berlin
- Brandenburg
- Bremen
- Hamburg
- Hessen
- Mecklenburg-Vorpommern
- Niedersachsen
- Nordrhein-Westfalen
- Rheinland-Pfalz
- Saarland
- Sachsen
- Sachsen-Anhalt
- Schleswig-Holstein
- Thüringen

14. Wieviele Einwohner hat Ihr Wohnort ungefähr?

- unter 500 Einwohner
- zwischen 501 und 5.000 Einwohner
- zwischen 5.001 und 20.000 Einwohner
- zwischen 20.001 und 100.000 Einwohner
- zwischen 100.001 und 500.000 Einwohner
- über 500.000 Einwohner

15. Wie alt sind Sie?

Ich bin ______ Jahre alt.

Zurück

Weiter

83% ausgefüllt

16. Welches Geschlecht haben Sie?

- weiblich
- männlich

17. Welchen Bildungsabschluss haben Sie?

Bitte wählen Sie den höchsten Bildungsabschluss, den Sie bisher erreicht haben.

- **Schule beendet ohne Abschluss**
- **Noch Schüler**
- **Volks-, Hauptschulabschluss**
- **Mittlere Reife, Realschul- oder gleichwertiger Abschluss**
- **Abgeschlossene Lehre**
- **Fachabitur, Fachhochschulreife**
- **Abitur, Hochschulreife**
- **Fachhochschul-/Hochschulabschluss**
- **Anderer**

18. In welcher Branche arbeiten/studieren Sie?

Bitte wählen sie zuerst die Branche aus, in der Sie Ihren Beruf ausüben, oder in der Sie sich in einer Ausbildung befinden. Danach klicken Sie bitte Ihre zugehörige Qualifikation an (auch Studenten).

- **Bau, Architektur, Vermessung**
- **Dienstleistung**
  - Hochschulabschluss in diesem Bereich
  - Aktuell im Studium
  - Abgeschlossene Ausbildung in diesem Bereich
  - Aktuell in Ausbildung
  - Keine Ausbildung in diesem Bereich
- **Elektro**
- **Gesellschafts-, Geisteswissenschaften**
- **Gesundheit, Medizin**
- **IT, Computer**
- **Kunst, Kultur, Gestaltung**
- **Landwirtschaft, Natur, Umwelt**
- **Medien**
- **Metall, Maschinenbau**
- **Naturwissenschaften**
- **Produktion, Fertigung**
- **Soziales, Pädagogik**
- **Technik, Technologiefelder**
- **Verkehr, Logistik**
- **Wirtschaft, Verwaltung**
- **Aktuell keine berufliche Tätigkeit (auch Schüler!)**
- **Sonstiges**

92% ausgefüllt

19. Bitte geben Sie Ihre E-Mail-Adresse ein, falls Sie am Gewinnspiel teilnehmen möchten. Diese wird getrennt von der Umfrage gespeichert und kann auch nicht nachträglich zugeordnet werden.

- [ ] Ich will am **Gewinnspiel** teilnehmen. Ich bin damit einverstanden, dass meine E-Mail-Adresse bis zur Ziehung der Gewinner gespeichert wird. Meine Angaben in dieser Befragung bleiben weiterhin anonym, meine E-Mail-Adresse wird nicht an Dritte weitergegeben.
- [ ] Ich interessiere mich für die **Ergebnisse dieser Studie** und hätte gerne eine Zusammenfassung per E-Mail.

20. Bitte geben Sie die nachfolgenden zwei erfundenen Wörter ein. Damit überprüfen wir, dass sich wirklich ein Mensch hinter dem Bildschirm befindet und nicht einfach nur eine Maschine den Bogen ausfüllt.

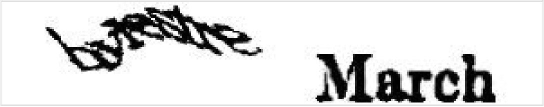

reCAPTCHA™

Zurück

Weiter

Danke für Ihre Teilnahme!

Wir möchten uns ganz herzlich für Ihre Mithilfe bedanken.

Falls wir Ihr Interesse an der Organspende geweckt haben, finden Sie weitere Informationen und natürlich auch die Möglichkeit einen Organspendeausweis zu erhalten, hier!

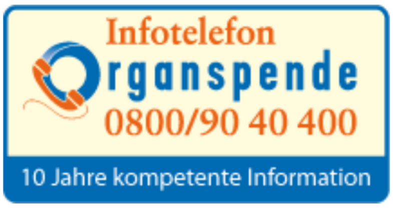

Supplement: Multimedia Appendix 1 [file ijmr_v4i3e16_app1.pdf]
