# Supplementary material for: Effectiveness of Organ Donation Information Campaigns in Germany: A Facebook Based Online Survey
Source: Interact J Med Res. 2015 Jul 28;4(3):e16. doi: 10.2196/ijmr.4287 (PMC4705356; doi:10.2196/ijmr.4287)
Supplement: Multimedia Appendix 2 [file ijmr_v4i3e16_app2.pdf]

Facebook Groups

ALLES EIN EURO

Alles für kleines Geld oder zum verschenken DORTMUND

Ausmisten macht Spaß Kreis Euskirchen

Babyflohmarkt online

Berlin

Bielefelder Flowmarkt

Biete zu verkaufen/verschenken

Biete/Suche/verkaufe/Verschenke rund um Aschaffenburg

Bis 5€ Gruppe

Business-Partner-Netzwerk

Charité Medizin SoSe 2010

Charité Modellstudiengang Medizin

Chiller Gruppe xd

Der DRESDEN City facebook Flohmarkt

Der Preis ist bis 2€ und nicht mehr

Deutsche TrainStation Gruppe

Die Besetzer

Die beste Adde-Gruppe

Die Fundgrube

Die größte Facebook Gruppe Deutschland´s

DIE Trödel Oase !

Dorfleben – suchtis Gruppe von ronny

Dortmund Kleinanzeigen

Dresden: suche-biete-tausche-verschenke…

Du & Ich

Du bist Unneraner , wenn …

Du lebst schon lange in Dortmund, wenn…

Du lebst schon langen in Kiel, wenn…

Du weißt, dass du aus Ulm/Neu-Ulm bist, . . .

Düsseldorfer Flohmarkt

Düsseldorfer Kleinanzeigen

Erstis 2012/13 Medizin Ulm

Erstis Uni Essen Medizin WS 12/13

Erstsemester Medizin Freiburg WS 11/12

Erstsemester Medizin Freiburg WS 12/13

Essener – Flohmarkt

EXPERIMENT: 25.000 Hundehalter in einer Gruppe? Ist das möglich?

Facebook An und Verkauf

Facebook Flohmarkt

FAHR DOCH SCHNELLER DU ARSCH!!!!! –wir fluchen im auto

Fanseiten Posten

FC Bayern München

Flensbook

Flohmarkt Bad Waldsee

Flohmarkt Dortmund

Flohmarkt Kreis Recklinghausen

Flohmarkt Rostock

FLOHMARKT TAUSCHBÖRSE MÜNSTER

Flohmarkt von A-Z

Flohmarktalarm :) werdet los was andere suchen :)

Frankfurt Medizin Klinik Erstis WS 10/11

Frankfurt Medizin Klinik Erstis WS 2012/13

Freunde Finden

Freundschaftsanfragen aus aller Welt. Hier darf jeder posten was er möchte.

Friends 4 me…Freundschaftsanfragen erwünscht.

Fundgrube Kamen

Garantiert kostenloses aus dem Internet

Getrennt in den Farben vereint in der Sache – Ruhrpott hält zusammen

Größte Facebook Gruppe

Grösste Facebook Gruppe ? Ladet alle ein !

Größte Gruppe der Welt ;D

Grösste Hip Hop Gruppe in Deutschland

Gruppe für Hundehalter

Gruppen Empfehlen

Gruppen Rekord 3.000.000 mitglieder bis zum 31.12.2012

Hab da was zu verkaufen…

Handy Verkaufen Tauschen ect.

Hartz4Flohmarkt

Hier können "Likes" abgeholt und vergeben warden!

Hier wird getauscht verkauft und geschenkt

Hilfst du mir, helfe ich Dir! :)

Ich habe schon Dinge über mich gehört, die wusst ich selbst noch nicht…

Ich suche ……..

Ich verkaufe………..Ich suche…………

iPad

iPad & iPhone in Deutsch

Karlsruher Flohmarkt & Sperrmüll

Kaufen – Verkaufen – Verschenken – Suchen

KAUFEN & VERKAUFEN….SUCHEN & FINDEN

Kaufen / Verkaufen / Tauschen / Bieten – hier ist alles möglich

Kaufen und verkaufen

Kaufen, Verkaufen

Kaufen,verkaufen und tauschen!

Kielbook

Kielder und andere Mamas Tauschen Kaufen und Verkaufen!!!

KIELER Flohmarkt

Kleinanzeigen : Bieten ,Suchen &Tauschen Düsseldorf

Kleinanzeigen „Dortmund und Umgebung"

Kleinanzeigen Kiel

Kohorte 14 – Medizin Köln

Köln

Kostet nix, alles umsonst !

Kunterbunter flohmarkt

Ladet alle eure freunde ein es muss die grösste Gruppe der Welt werden :D

Ladet alle eure freunde ein und werdet mitglied der größten gruppe

Leipziger Online Flohmarkt

Lokalkompass Niederberg

Magdeburg Medizin Erstis 2012/2013

Mainz Erstis Medizin SoSe 2011 und WiSe 10/11

Mainz Erstis Medizin Wintersemester 2012/13

Mainz Erstis Medizin WiSe 2011

Medizin Aachen Gruppe 1 ab WS 12/13

Medizin Erstis Bonn WS 2009/2010

Medizin Erstis Bonn WS 2010/11

Medizin Erstis Erlangen SS 13

Medizin Erstis Erlangen SS` 12

Medizin Erstis Erlangen WS' 12/13

Medizin Gießen SS 2010

Medizin Gießen WS08/09

Medizin Heidelberg 08

Medizin Jena 2008

Medizin Jena 2009

Medizin Köln SS 2013

Medizin Marburg WS 2012/13

Facebook Groups

Medizin Würzburg SoSe 2013

Medizin-Giessen 2011

Meidzin WS 11/12 in Gießen

Meine Freunde und ihre Freunde ;) auf eine riesen Gruppe ;)

Mit Werbung zum Erfolg

Modellstudiengang Medizin Bochum

Mona´s Verkaufsecke

München verschenkt

Neuer GruppenWeltrekord – schaffen wir die 2 Millionen ?

Neus-Kleinanzeigen

Neuss Kleinanzeigen

Neusser Flohmarkt

Nicki`s Flohmarktkiste

Nur zu Verschenken ! Kein Verkauf!!!

Oberberger kleinanzeiger!

Online Flohmarkt GÖPPINGEN & Umgebung

Online Flohmarkt Hannover und Umgebung

Party-People Germany Gruppe

Pottkinder, Tradition Ruhrpott

QUERDENKER-Club

Rammstein Universum

Regensburger Erstsemester Medizin WS2012/13

Regensburger HOPSis – Medizin 2011

Rostocker Medizinstudenten 2012

Sachen zu Verkaufen oder Verschenken in Minden

Schafen wir 100.000 Mitglieder in einer Gruppe?!

Schenken und Geschenktes suchen

Schenken-Tauschen – Suchen

Schwarzes Brett – FH Aschaffenburg

SecondHandBörse

Stuttgart for free

Stuttgart verschenkt!

Stuttgarter helfen Stuttgarter

Suche- Tausche- Multimedia In Korbach

Suche und biete an

SUCHE und BIETE in Kiel & Umgebung…

Suche, Kaufe, Verkaufe, Tausche, die FB Börse!!

Suche,Verkaufe,Verschenke

Suchen – Finden – Bieten – Kaufen – Verkaufen :D

SUCHEN/ZU VERKAUFEN/VERSCHENKEN

Suchen….Verkaufen…Verschenken oder Tauschen

Tauschbörse…Hier geht alles,tauschen,bieten,verkaufen oder versteigern :)

Tauschen , Kaufen , Verkaufen , GRATIS in ÖSTERREICH

ThermoNixen-die Gruppe die süchtig macht!

TOP ELEVEN GRUPPE

Trödel & Schnäppchen

TSG 1899 Hoffenheim

Undergrunde Hopper

Uni Bonn: Medizin – Vesalius Semester

Uni Essen – Medizin – Meyer-Schwickerath-Semester (WS12/13)

Velbert An un Verkauf

VERKAUF: Alles rund um den Haushalt und auch sonst noch einiges!

Verkaufe aschaffenburg

Verkaufe,Suche…Verschenke!

Verkaufe/Suche Sinsheim

Verkaufen, tauschen, verschenken

Verkaufen,Tauschen,Suchen .Fürth und Umgebung

Verkaufs und Flohmarkt Gruppe Schleswig-Holstein

Verschenk ´s Erfurt

Verschenk ´s- Halle

Verschenke/Günstig abgeben.

Verschenken, verkaufen,gesucht

Vertick dein Zeug!

Vertriebs – u. Verkaufsportal

Virtuelle Flohmarkt Bremen, Bremerhaven, Hamburg, Hannover und umgebung

WER NICHT WIRBT – DER STIRBT (Werbe-Gruppe für Alle & Alles)

Werbung für alle !!!

Wir sind Hier – Die Gruppe

Wir Verschenken gegen Porto

Zu verkaufen/verschenken

Zu Verkaufen

Zu Verkaufen & Verschenken

Zu Verkaufen,zu verschenken und Suche und Biete im Raum Meiningen

Zu verschenken

Zu verschenken

Zu verschenken !! Schenken statt wegwerfen :)

Zu verschenken in VS, TUT und RW

Zu verschenken Neuss/Düsseldorf

Zu Verschenken!!!!

2.000.000.000

Zauberland

1.000.000 Ladet alle eure Freunde ein ;)

1.000.000 Mio. Mitglieder ladet alle eure Freunde ein

1.000.000 Mitglieder bis 31.12.2013 , 23.59 Uhr ! Macht mit !

10.000 mitglieder

10.000.000 Menschen?

2 Tauschen und Verschenken,Verkaufen.Kinder,Frauen u Herren Klamotten.

4. Semester Medizin Uni Mainz
