# Supplementary material for: Effectiveness of Organ Donation Information Campaigns in Germany: A Facebook Based Online Survey
Source: Interact J Med Res. 2015 Jul 28;4(3):e16. doi: 10.2196/ijmr.4287 (PMC4705356; doi:10.2196/ijmr.4287)
Supplement: Multimedia Appendix 3 [file ijmr_v4i3e16_app3.pdf]

Literature of educational interventions on organ donation and their results

Author	Year	Country	Method	N	Target Group	Results
Heuer et al.	2014	Germany	Two-piece questionnaire with an informational section between the questionnaires.	1818	Employees of the University Duisburg-Essen	"Of the interviewees who read the information, 88% stated that such information was sufficient for their needs; [...] Of interviewees who initially stated that they objected to an organ donation for themselves, 20% (n = 196) supported organ donation after reading the information. [...] Two thirds of the interviewees (66.6%) who did not yet possess an organ donor card were positively inclined to hold one in the future."
Rey et al.	2013	Germany	Questionnaire-based survey. All students were offered an information event. The survey was repeated later.	1164 (first survey), 1491 (second survey)	11th and 12th-grade students of secondary schools	"The second survey showed that the percentage of donor card carriers had nearly doubled as compared to the first survey (11.3% vs. 19.6%)." "More often than others students, those who approved organ donation had talked about organ donation in the family (OR 1.75, 95% CI: 1.35–2.27), had sought information about organ donation within the last 12 months (OR 5.95, CI: 3.61–9.81)"
Yilmaz et al.	2011	Turkey	Questionnaire-based survey. The researcher gave a lesson and then collected the data by the same questionnaire 2 months later.	132 (first survey), 128 (second survey)	Soldiers in a military unit	"The number of participants willing to donate their organs increased to 112 (87.5%). [...] (P < .0001)." "The rate of volunteering to donate whole organs increased significantly after the lesson (P < .0001).[...] religious beliefs decreased significantly (P < .0001)." "Volunteering increased significantly (P < .0001)."
López-Montesinos et al.	2010	Spain	32-hour training course about donation and transplantation. Survey before and after the course.	48	Students in the third year of nursing	"Attitude toward organ donation after death was favorable in 87% of respondents, increasing to 94% after course completion, although there was not a significant difference (P = .278)."
Frates et al.	2006	USA	Paid television and radio campaign to promote organ and tissue donation within the Hispanic community. Survey once prior to the campaign and during each of the 3 project years.	500	Hispanic Americans	"The consent rate for organ donation [...] for Project Years Two and Three [...] exceeded project objectives. (Increase the number of Hispanic individual declarations of intent to donate organs in the agency's service area by 5% in Project Year Two and 10% in Project Year Three)[…] Measures of attitude and behavior met or exceeded project objectives in Project Year Two (2002), then declined in Year Three (2003)."
Smits et al.	2006	Netherlands	Students were randomly assigned to a control group or to an experimental group who received a 45-min lesson about organ donation.<br>Both groups filled in a questionnaire.	319	Secondary school students	"The experimental group and control group differed significantly in their intention to register […]P < 0.001." "More students from the intervention group reported to be willing to donate organs posthumously.[...]A larger proportion of the students in this group did not want to donate organs after death in comparison with the control group. More members of the control group would not send back the form and would leave the decision to others (35.6% versus 20.4%). The lesson [...] appeared to enabled students to make a decision themselves."
Tokalak et al.	2006	Turkey	Students were surveyed with a questionnaire. Then, the students underwent a training program on organ donation and transplantation. Then, they filled out the same questionnaire again.	189 (first survey), 138 (second survey)	High school students	"Prior to the training program, 24.9% of the 189 respondents stated that they were willing to donate their organs after death, whereas after training 38.4% of the 138 respondents said they would do so (p<0.001). Before training, 14.3% of the respondents said they were absolutely opposed to organ donation, and this rate fell significantly to 7.2% after the program was completed (p<0.001)."
Reubsaet et al.	2005	Netherlands	Students were randomly allocated to either attend a organ donation education program or not. Then they filled out a questionnaire.	2868	High school students	"Students in the experimental group had significantly higher measures of intention to register their organ donation preference and more often intended to register as a posthumous organ donor. Further, on average, students who were exposed to the lessons answered four more knowledge items correctly (item range: 0–26) compared to students who were not exposed to the lessons. Students in the experimental group experienced greater registration self-efficacy, fewer negative outcome expectations and more positive social outcome expectations regarding organ donation registration than students in the control group."
Piccoli et al.	2004	Italy	high schools in were randomized as interventions (n =7) or controls (n=7).<br>Intervention group:<br>1. questionnaire,<br>2. intervention,<br>3. questionnaire.<br>Control group: 2 Questionnaires	intervention: 937 first and 808 second questionnaires;<br><br>controls: 739 and 659	High school students	"The opinions on cadaveric transplantation varied: in the control schools, no difference was found between first and second questionnaires; while among the intervention schools there was a significant increase in favorable and uncertain opinions and a decrease of negative ones[...] (P < .05)."
Rachmani	2000	Israel	1. questionnaire, 2. two-day-workshop, 3. the same questionnaire	86 doctors,<br>90 nurses	personnel of departments relevant to organ donation	"The findings revealed significant differences in all the indexes between the knowledge, attitude, and behavioral intention—before and after the workshop. Significant differences were found also in the knowledge and attitudes toward the three main stages in the organ donation process before and after the workshop."
Weber, Napieralski	1999	Germany	Intervention group: Two identical questionnaires, one before and one after the lectures.<br>Control group: Questionnaire	185 (Intervention group),<br>1257 (control group)	Members of seven lectures about organ donation (Intervention group)	"[...] the main target issue (ie, the willingness to donate organs after death) did not change as a result of the lecture, nor did the wish to be transplanted. From those who offered a statement in regard to the main target question in both questionnaires (N=180) 19 respondents changed their mind more in opposition to and 56 more in favor of organ donation (on the scale from 1 to 6). All others remained unimpressed by the presentation."
Weber et al.	1999	Germany	Two questionnaires (1 in 1994, 1 in 1998). Participants have been asked wether they had followed at least one television discussion about organ donation (intervention).	940 (1994), 756 (1998)	High school students' parents	"The difference in the answers of TV+ and TV- respondents to [Do you have a donor card?] did not reach the significance level, however, there was a trend towards more owners of donor cards in the TV+ group."<br>"[...]There were no significant differences between TV groups [...] with regard to [Would you agree to organ donation in case of your death?][...]"
Wig et al.	1999	India	Questionnaires to assess knowledge of brain death and organ transplantation. Then they were provided with educational information about the topic. This was followed by similar questionnaires.	188	High school students	"Post-education, there was significant increase in awareness of specific organ transplantation. Awareness about various aspects of brain death increased remarkably after education[...]. Majority of students (98%) were able to understand the concept of brain death. All of them agreed that life cannot return after brain death and that brain death is synonymous with death. Most of them (95%) agreed with the concept of brain death and 85% were of the view that their religious background allows organ donation."

Based on Pubmed search "organ donation" AND survey AND information AND knowledge (May 2015)
