# Supplementary material for: Effectiveness of Organ Donation Information Campaigns in Germany: A Facebook Based Online Survey
Source: Interact J Med Res. 2015 Jul 28;4(3):e16. doi: 10.2196/ijmr.4287 (PMC4705356; doi:10.2196/ijmr.4287)
Supplement: Multimedia Appendix 4 [file ijmr_v4i3e16_app4.pdf]

Literature of the correlation between knowledge about organ donation and the willingness to donate

Author	Year	Country	Method	N	Target Group	Results	Annotations
Febrero et al.	2014	Spain	self-administered anonymous questionnaire	288	Secondary school teacher	"Those who reported having very good or good information were more in favor [towards organ donation] than those who had limited information or none at all (88% vs. 61%, respectively; p=0.001)."	*See Febrero et al. 2013*
Ali et al.	2013	Pakistan	self-administered anonymous questionnaire	158	Medical students	"A very highly significant association (P=0.000) was found between willingness to donate and knowledge of allowance of organ donation in religion."	
Tumin et al.	2013	Malaysia	interviewer-administered questionnaire	779	Malaysian muslims	"The majority of the respondents who said 'yes' to organ donation but did not register did so because they were not aware of the organ donation procedures."	
Febrero et al.	2013	Spain	self-administered anonymous questionnaire	288	Secondary school teacher	"Those who reported having good or very good information were more in favor of [organ donation and transplantation] (P < .001)."	*See Febrero et al. 2014*
Cucchetti et al.	2012	Italy	self-administered anonymous questionnaire	2258	Italian Facebook users	"Know the significance of brain death significantly affected both being in favour of donation (P=0.020) and consent to donation (P<0.001)"	
Rey et al.	2012	Germany	self-administered anonymous questionnaire	1155	Secondary school students	"37.0% of the students declined organ donation. Of these, 72.4% named a lack of education and informations as the primary reason for this statement."	
Ríos et al.	2012	Spain	self-administered anonymous questionnaire	288	Secondary school teacher	"The respondents who knew the concept of [brain death] were more in favor of deceased donation than those who did not understand the concept or who had doubts (81% versus 65%; P <.01)."	
Trompeta et al.	2012	USA	self-administered questionnaire	121	Asian American adolescents living on the Big Island of Hawaii and their parents	"Willingness to donate was associated with positive knowledge related to general organ donation and barriers, low level of opposing attitude about organ donation, and a higher acculturation level"	
Ríos et al.	2011	Spain	self-administered anonymous questionnaire	1611	British and Irish residents in southeast Spain	"There was a close relationship between attitude and understanding of the concept of [brain death]. [Of] those who were in favor of deceased donation, 49% understood the concept of BD, while only 36% of those who were not in favor understood the concept (P<0.001)."	
Wong	2011	Malaysia	computer-assisted telephone interview	1174	Malaysian adults	"Participants who reported that they have registered to be organ donors, knew where to register to be organ donors, and willing to donate one's own and loved-one's organs recorded significantly higher mean total knowledge score."	
Al-Ghanim	2009	Saudi Arabia	self-administered questionnaire	487	University students	"Students who had information on brain death, and donation card, and knew someone who had donated, or had discussed the issue with others were more likely to consent to deceased organ/tissue donation"	
Saleem et al.	2009	Pakistan	interviewer-administered questionnaire	408	Pedestrians at market places of Karachi	"[…]knowledge scores for organ donation were significantly associated with the motivation to donate an organ (p = 0.002)."	
Ríos et al.	2008	Spain	self-administered anonymous questionnaire	414	Primary health care personnel	"[…] 62% (n = 255) of respondents understand the concept of brain death and this understanding is significantly associated with attitude toward donation (p = 0.000)."	
Conesa et al.	2005	Spain	self-administered questionnaire	2000	People > 15 years of age	"A statistical association has been observed between favourable public opinion and different psychosocial variables (p < 0.05), with some independent variables persisting in the multivariate analysis such as [...] knowledge of the concept of brain death (OR = 1.4)"	
Gross et al.	2001	Switzerland	self-administered questionnaire	7272	Swiss-Italian recruits	"A significantly higher agreement to donate their organs was found among men […] who felt they were sufficiently informed about the subject of transplantation (p <0.0001)"	
Pugliese et al.	2001	Italy	self-administered questionnaire	1576	Staff working in areas involved in organ procurement and transplantation	"In favor of organ donation were 96% physicians, 86% nurses, and 82% from other backgrounds (P < 0.001). These percentages decrease to 85% for physicians, 76% for nurses, and 69% for others, when asked about their willingness to donate their own organs (P < 0.0001)."	
Saub et al.	1998	USA	self-administered questionnaire	378	Volunteers from the Orange County, California Superior Court jury pool	"those who thought they knew how to make arrangements to donate were more than four (unadjusted odds ratio) times as likely to donate compared with those who did not know how to make the arrangements to donate."	

Based on Pubmed search "organ donation" AND survey AND information AND knowledge (May 2015)
